# PLAAT1 inhibits type I interferon response *via* degradation of IRF3 and IRF7 in Zebrafish

**DOI:** 10.3389/fimmu.2022.979919

**Published:** 2022-09-12

**Authors:** Xin Zhao, Wenji Huang, Yanjie Shi, Jiahong Guo, Hehe Xiao, Ning Ji, Jianhua Feng, Huifeng Dang, Jun Zou

**Affiliations:** ^1^ Key Laboratory of Exploration and Utilization of Aquatic Genetic Resources, Ministry of Education, Shanghai Ocean University, Shanghai, China; ^2^ International Research Center for Marine Biosciences, Ministry of Science and Technology, Shanghai Ocean University, Shanghai, China; ^3^ National Demonstration Center for Experimental Fisheries Science Education, Shanghai Ocean University, Shanghai, China; ^4^ Laboratory for Marine Biology and Biotechnology, Qingdao National Laboratory for Marine Science and Technology, Qingdao, China

**Keywords:** PLAAT1, IRF3, IRF7, interferon, autophagy, virus

## Abstract

PLAAT1 is a member of the PLAAT protein family and plays important roles in tumor suppression, transglutaminase activation and peroxisomal biogenesis. Recently, PLAAT1 has been shown to promote degradation of p53 protein and cellular organelles such as mitochondria, endoplasmic reticulum and lysosome. In this study, we show that PLAAT1 inhibits the production of type I interferon and promotes virus replication in zebrafish. Overexpression of *Plaat1* in zebrafish cells suppresses antiviral responses and promotes virus replication. Mechanistically, PLAAT1 interacts with IRF3 and IRF7 to initiate degradation of IRF3 and IRF7, which can be attenuated by 3-methyladenine, an inhibitor of autophagosome. Our study provides novel insights into the functions of PLAAT1 in host immune response to viral infection.

## Introduction

Detection of pathogen-associated molecular patterns (PAMPs) by host pattern-recognition receptors (PRRs) induces interferon (IFN) production, triggering cellular responses that confer to the protection against virus infection (1). In fish, a large panel of evolutionarily conserved PRRs have been characterized, including the RIG-I-like receptors (RLRs) and Toll-like receptors (TLRs) and cytosolic DNA sensors (2, 3). RLRs including RIG-I and melanoma differentiation-associated gene 5 (MDA5) and TLRs such as TLR3, TLR7 and TLR8 can sense viral RNA to induce MAVS-mediated IFN production (4–7). DNA sensors such as TLR9 and cGAS detect cytosolic viral DNA to activate IFN response through the adaptor protein STING (8–10). Both MAVS-dependent and STING-dependent signaling cascades lead to the recruitment and activation of TANK-binding kinase 1 (TBK1) and subsequent phosphorylation and dimerization of the interferon-regulatory factors (e.g. IRF3 and IRF7). Interferons bind to a heterodimeric receptor to induce the expression of hundreds of interferon-stimulated genes (ISGs) to defend viruses (11).

Interferon-regulatory factors (IRFs) belong to a family of transcription factors which regulate the production of type I IFNs and host antiviral response (12, 13). The IRF family consists of 9 members in mammals and 11 members in fish (14). They all contain a conserved N-terminal DNA-binding domain (DBD) which recognize and specifically bind to DNA motifs similar in sequence to the IFN-stimulated response element (ISRE). The C-terminal region contains an IRF-associated domain (IAD) responsible for the interaction with members of the IRF family and/or other factors (15). Among IRF members, IRF3 and IRF7 are closely related and harbor a serine-rich region in their C terminus which is critical for the virus-induced phosphorylation events and downstream signaling (16–19). In fish, the structure and functions of IRF3 and IRF7 are well conserved.

PLAAT1 was originally identified as a tumor suppressor and termed A-C1 (20). PLAAT1 is a phospholipase A_1/2_ and belongs to the phospholipase A/acyltransferase (PLAAT) family. The PLAAT proteins are evolutionarily conserved in vertebrates and have a broad substrate specificity towards a range of glycerophospholipids (21–23). They all share 4 conserved domains: a proline-rich motif at the N-terminus, a conserved H-box, an NC motif (NCXHFV) and a C-terminal transmembrane domain (24). In mammals, the PLAAT family consists of five members, PLAAT1-5, and all are multifunctional enzymes that possess N- and O- acyltransferase and phospholipase_1/2_ activity (23, 25, 26). They play important roles in regulating tumor progression, transglutaminase activation, peroxisomal biogenesis and virus entry into the host cells (21, 27, 28). PLAAT3, also termed PLA2G16, is an adipose-specific phospholipase A2 and serves as a host factor to uncoat enterovirus genome into the cytoplasm of target cells (29). PLAAT3 has also been shown to promote degradation of nuclear DNA by damaging the nuclear envelope and/or enhancing the release of lysosomal DNase 2B (30–32). When overexpressed in the HEK293 cells, PLAAT3 reduces the number of peroxisomes by inhibiting peroxisome biogenesis through interaction with PEX1921 (27). Recently, PLAAT1 has been shown to be required for the degradation of cellular organelles such as mitochondria, endoplasmic reticulum and lysosome (33). Giving the importance of the cellular organelles in host immune response and virus replication, we reason that PLAAT1 may play a role in the interaction between host and viruses.

Previously, we showed that overexpression of *plaat1* in zebrafish cells inhibited mRNA expression of *p53* and *tnf-α*, and induced degradation of p53 (34). In this study, we investigated the functions of PLAAT1 in host immune responses to viral infection in zebrafish. We found that zebrafish PLAAT1 interacted with IRF3 and IRF7 and promoted their degradation, resulting in suppression of type I IFN production.

## Materials and methods

### Cells and viruses

ZF4 and EPC cells were cultured at 28 °C in a 5% CO_2_ incubator in Dulbecco’s modified Eagle’s medium (DMEM, Gibco) supplemented with 10% fetal bovine serum (FBS, Gibco). Spring viremia of carp virus (SVCV), provided by Dr Mingxian Chang, was propagated in the EPC cells. Virus titer was determined based on the 50% tissue culture infective dose (TCID_50_) assay.

### Plasmids and antibodies

Full-length cDNA of zebrafish *plaat1* was cloned into pcDNA3.1 with a Flag tag and pEGFP-N1 (34). Synthetic full-length cDNA fragments of zebrafish *irf3* and *irf7* (GENEWIZ, China) were cloned into pcDNA3.1 with a Myc tag at the C terminus, respectively.

Antibodies used in this study included α-Flag (Huabio, China), α-Myc (Huabio, China), α-*β*-actin (Huabio, China), α-GFP (Zen-bio, China), α-GFP (Abmart, UK), α-mouse IgG (LI-COR, USA) and α-rabbit IgG (LI-COR, USA) Abs. Following reagents were used: TRIzol (Thermo Fisher, USA), Hifair^®^ 1st Strand cDNA Synthesis SuperMix (Yeasen, China), Hieff UNICON^®^ Power qPCR SYBR Green Master Mix (Yeasen, China), MG132 (Aladdin, USA), 3-methyladenine (3-MA) (Aladdin, USA), chloroquine (CQ) (Sigma-Aldrich, USA), polyinosinic:polycytidylic acid [poly(I:C)] (Sigma-Aldrich, USA), protein A/G resin (Yeasen, China), radioimmunoprecipitation assay (RIPA) buffer (Beyotime, China), and JetOPTIMUS plasmid transfection kit (Polyplus, China).

### Quantitative real-time PCR

Total RNA was extracted using TRIzol reagent according to the manufacturer’s protocol. Quantitative real-time PCR (qRT-PCR) was run in triplicate on a Light Cycler^®^ 480 Real-Time PCR System (Roche, Switzerland). The qRT-PCR reactions were set up as follows: 5 μL Hieff UNICON^®^ qPCR SYBR Green Master Mix, 1 μL cDNA template, 0.2 μL forward primer (10 μM), 0.2 μL reverse primer (10 μM) and 3.6 μL distilled water, and run under following conditions: 1 cycle of 95 °C for 30 s, 40 cycles of 95°C for 5 s, 62°C for 30 s, 72°C for 10 s, followed by 1 cycle of 95°C for 10 s, 65°C for 60 s, 97°C for 1 s. The qRT-PCR primers are listed in **Table 1**. The elongation factor alpha (*ef1α*) was used as internal control to normalize gene expression.

**Table 1:** Primers used in this study.

Primer name	Sequence (5’→3’)
RT-*ef1α*-F	CTGGAGGCCAGCTCAAACAT
RT-*ef1α*-R	ATCAAGAAGAGTAGTACCGCTAGCATTAC
RT- *ifnφ1*-F	TGGAGGACCAGGTGAAGTT
RT- *ifnφ1*-R	ATTGACCCTTGCGTTGCTT
RT-*SVCV n*-F	TCTGCCAAATCACCATACTCA
RT-*SVCV n*-R	CTGTCTTGCGTTCAGTGCTC
RT-*SVCV g*-F	ATCATTCAAAGGATTGCATCAG
RT-*SVCV g*-R	CATATGGCTCTAAATGAACAGAA
RT-*plaat1*-F	AGTCGGTGTTCAGCCGTAAAG
RT-*plaat1*-R	TGACGAAGTGCTCACAGTTGC

F, forward; R, reverse.

### Luciferase assay

EPC cells were seeded in 24-well plates and transfected with a mixture of 250 ng of luciferase reporter (firefly luciferase) and 25 ng of pRL-TK (Renilla luciferase plasmid), together with reporter plasmids or vector plasmid. At 24 h post transfection, the cells were transfected with poly(I:C) or infected with SVCV and cultured for 24 h before harvest. The cells were lysed for measuring luciferase activity using the Dual-Luciferase Reporter Assay System (Promega, USA) according to the manufacturer’s protocol. As for the IFN/ISRE promoter activation assay, various plasmids at a ratio of 10:10:10:1 (expression vectors of irf3/irf7/IFNφ1pro/ISRE-Luc/pRL-TK) were used for transfection. Empty vector pcDNA3.1 was used to ensure that there were equivalent amounts of total plasmid DNA for transfection. Firefly luciferase activity was normalized based on the Renilla luciferase activity.

### Western blotting

Cultured cells were washed with ice-cold PBS and lysed in RIPA buffer (Beyotime, China) supplemented with 1 mM phenylmethylsulfonyl fluoride (PMSF) and protease inhibitor cocktails (1:100, v/v, Beyotime, China). Protein solution or whole cell lysates were resolved in SDS-PAGE loading buffer (Sigma-Aldrich, USA), transferred to a polyvinylidene difluoride (PVDF) membrane (Millipore, USA), and probed with the primary and secondary antibodies. Western blotting images were photographed using an Odyssey CLx Imaging System (LI-COR, USA).

### Co-immunoprecipitation

HEK293 cells were seeded in 25-cm^2^ flasks and cultured overnight. The cells were transfected with PLAAT1-GFP (5 *μ*g) and Myc-IRF3 (5 *μ*g) or Myc-IRF7 (5 *μ*g) plasmids. At 24 h post transfection, the adherent cells were washed twice with ice-cold PBS and covered with RIPA lysis buffer containing protease inhibitor cocktails. The cell culture flasks were placed on a rocker platform and rotated at a slow speed at 4°C for 30 min. The lysate supernatants were collected by centrifugation at 12,000 x *g* at 4°C for 15 min and incubated with 35 *μ*L α-GFP affinity gel (Abmart, UK) at 4°C overnight. Immunoprecipitated proteins were collected by centrifugation at 2,000 x g for 3 min, washed three times with ice-cold PBS, and resuspended in 50 *μ*L of 2 x SDS-PAGE sample buffer and then subjected to SDS-PAGE and Western blotting.

### Fluorescent microscopy

EPC cells were cultured on coverslips in 6-well plates and transfected with 1 *μ*g plasmid and cultured for 24 h. The cells were washed twice with PBS, fixed with 4% Paraformaldehyde Fix Solution (PFA) (Beyotime, China) for 10 min, washed three times with PBS, and blocked with PBS containing 5% BSA for 1 h. The cells on coverslips were incubated with the primary antibody at 4°C overnight, followed by incubation with Alexa Fluor 594-conjugated α-rabbit IgG (Cell Signaling Technology, USA) or Alexa Fluor 594-conjugate α-mouse IgG (Zenbio, USA) for 1 h. The cells were washed three times with PBS and stained with 1 *μ*g/mL 4,6-diamidino-2-phenylindole (DAPI, Beyotime, China) for 10 min in the dark at room temperature. The coverslips were then washed, examined under a Leica confocal microscope (Leica SP8) and photographed.

### Statistical analysis

The qRT-PCR data were analyzed using the SPSS package 20.0 (SPSS Inc., Chicago, IL, USA) and One-way ANOVA and the LSD *post hoc* test. “*p <0.05” between treatment groups and the corresponding control groups are considered significant.

## Results

### PLAAT1 is not modulated at the transcription level by poly(I:C) or SVCV

**Figure 1:**
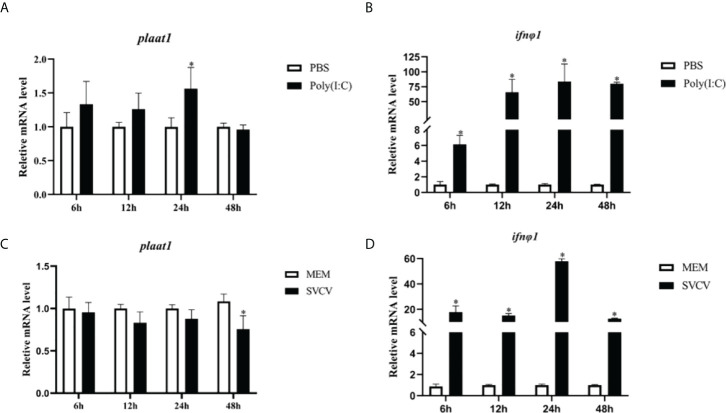
*Plaat1* expression in ZF4 cells in response to poly(I:C) and SVCV infection. ZF4 cells were seeded in 6-well plates and cultured overnight. The cells were transfected with poly(I·C) (2 *μ*g) or infected with SVCV (3.16 × 10^6^ TCID_50_/mL). Gene expression of *plaat1* **(A, C)** and *ifnφ1* **(B, D)** was analyzed by qRT-PCR. Data are presented as mean ± SEM, n=3. *P<0.05 is considered significant difference.

Previously, we found that PLAAT1 promotes p53 degradation and plays a regulatory role in p53 mediated signaling and autophagy. It has been reported that p53 is involved in a wide range of cellular responses including immune response to infection. To examine the transcriptomic response of *plaat1* in antiviral response, ZF4 cells were transfected with poly(I:C) or infected with SVCV. As shown in **Figures 1A, C**, the mRNA levels of *plaat1* were generally unaffected by poly(I:C) transfection and SVCV infection. However, the *plaat1* gene was marginally induced 24 h after stimulation with poly(I:C) but downregulated at 48 h following SVCV infection. As expected, the expression of *ifnφ1* was significantly upregulated (**Figures 1B, D**).

### PLAAT1 represses IFN expression induced by poly (I:C) and SVCV

**Figure 2:**
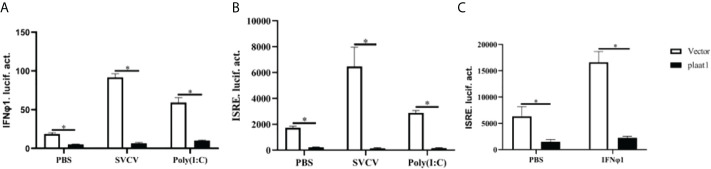
Zebrafish PLAAT1 negatively regulates IFN expression and signaling. **(A, B)** Overexpression of *plaat1* suppressed the activity of IFNφ1 **(A)** or ISRE **(B)** luciferase reporter induced by SVCV infection or poly (I:C). EPC cells were seeded in 24-well plates and transfected with IFNφ1pro-Luc (250 ng) or ISRE-Luc (250 ng) plus pRL-TK (25 ng) and Flag-*plaat1* or pcDNA-3.1 (empty vector, 250 ng). After 24 h, the cells were infected with SVCV (3.16 × 10^6^ TCID_50_/mL) or transfected with poly (I:C) (2 *μ*g/mL). After 24 h, luciferase reporter activity was analyzed. **(C)** The EPC cells were transfected with ISRE-Luc (250 ng) plus pRL-TK (25 ng) and Flag-*plaat1*or pcDNA-3.1 (empty vector, 250 ng). At 24 h post-transfection, cells were treated with PBS or IFNφ1 protein (100 ng/mL) for 6 h. Data are shown as mean ± SEM (n=3). *P<0.05 is considered significant difference.

Although we did not observe modulatory effects of poly(I:C) and SVCV on the expression of *plaat1* at the transcription level, we sought to investigate whether PLAAT1 is involved in mediating IFN mediated response at the protein level, which is central for the antiviral defense. We overexpressed Flag-*plaat1* in the EPC cells and assessed the promoter activities of IFNφ1 and IFN stimulating response element (ISRE) following stimulation with poly(I:C) or infection with SVCV. It is apparent that overexpression of Flag-*plaat1* suppressed the activation of the IFNφ1 promoter (**Figure 2A**). Consistently, the ISRE activity was also inhibited after treatment with poly(I:C) or infection with SVCV (**Figure 2B**). Further, we examined the effect of *plaat1* on the ISRE promoter activity after stimulation with the recombinant IFNφ1 protein (produced in our laboratory). We found that the activation of the ISRE promoter was significantly enhanced and this IFN induced effect was attenuated by *plaat1* overexpression (**Figure 2C**). The ISRE motifs are conserved binding sites for the ISGs that respond to IFN activation. These results suggest that PLAAT1 acts as a negative regulator for IFN production and IFN mediated signaling.

### Overexpression of *Plaat1* increases SVCV replication in the EPC cells

**Figure 3:**
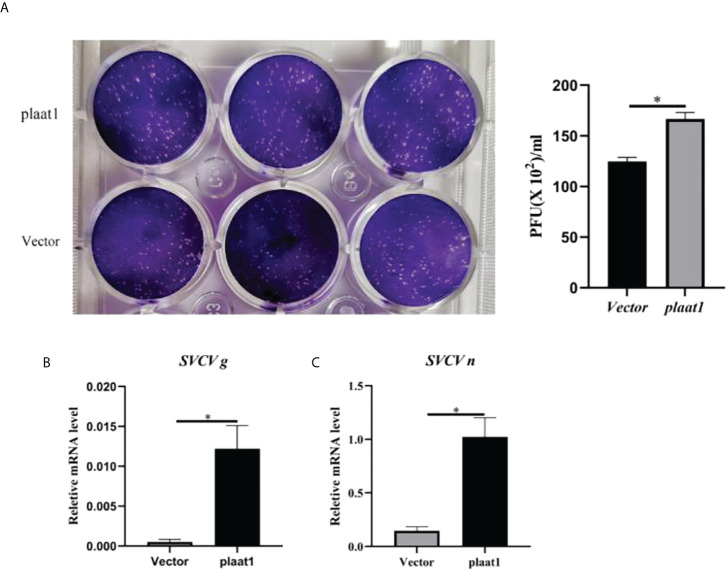
Overexpression of *plaat1* promotes SVCV replication in the EPC cells. The EPC cells were transfected with 2 *μ*g of Flag-*plaat1* plasmid or empty vector (pcDNA3.1). At 24 h post-transfection, cells were infected with SVCV (3.16 × 10^6^ TCID_50_/mL) for 24 h. Cell culture media were collected for plaque assay **(A)**. The mRNA levels of the *SVCV g* **(B)** and *SVCV n* **(C)** genes were analyzed by qRT-PCR. Data are shown as mean ± SEM (n=3). *P<0.05 is considered significant difference.

Since PLAAT1 inhibited the promoter activity of IFNφ1 and ISRE, we sought to perform plaque assay to assess the viruses released to the culture media. The culture media were collected from the *plaat1*-overexpressing EPC cells following infection with SVCV and used for plaque assay. Compared with the control cells (transfected with pcDNA3.1), higher numbers of viruses were detected in the culture media of *plaat1*-overexpressing EPC cells (**Figure 3A**). Consistently, the mRNA expression levels of the *SVCV g* (glycoprotein) and *n* (nucleoprotein) genes were markedly increased in the *plaat1*-overexpressing EPC cells (**Figures 3B, C**). These data suggest that zebrafish PLAAT1 promotes replication of SVCV.

### PLAAT1 targets IRF3 and IRF7 to suppress IFN response

**Figure 4:**
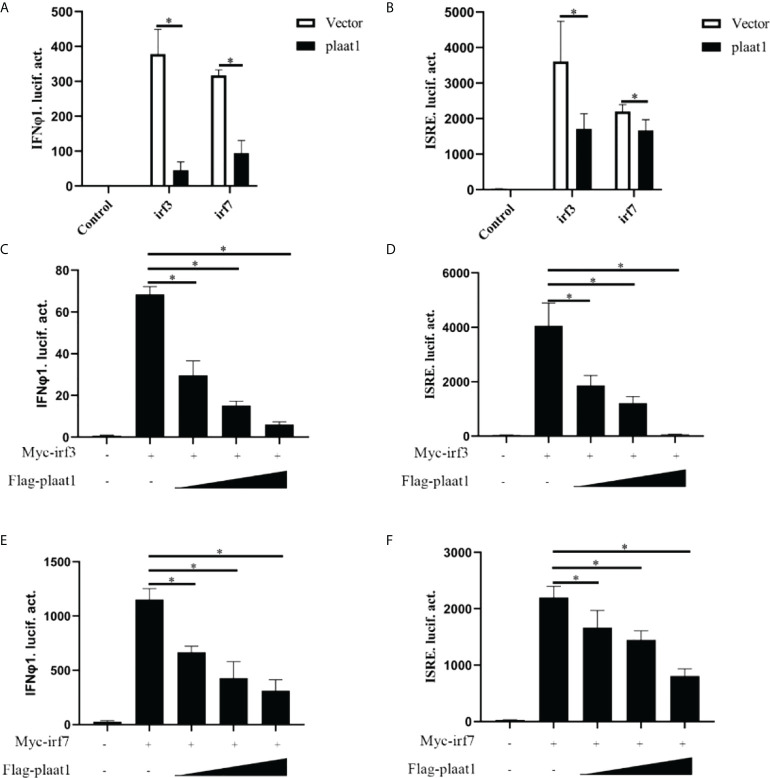
PLAAT1 targets IRF3 and IRF7 to suppress IFN response. **(A, B)** Overexpression of *plaat1* inhibits the activation of IFNφ1/ISRE promoters induced by *irf3* and *irf7*. EPC cells were transfected with indicated plasmids and collected at 24 h for luciferase assay. **(C–F)** Different doses of *plaat1* plasmid (250, 500, or 1000 ng) were used for transfection. Data are expressed as mean ± SEM (n=3). Data are shown as mean ± SEM (n=3). *P<0.05 is considered significant difference.

IRF3 and IRF7 are the master transcription factors to control IFN production (35). Upon activation of PRRs by viral pathogen associated molecular patterns (PAMPs), IRF3 and IRF7 are phosphorylated in the cytoplasm, form homo- or hetro- dimers and subsequently translocate into the nucleus to activate IFN expression. We reasoned that PLAAT1 may target IRF3 and IRF7 to inhibit IFN response. We analyzed the activity of IFNφ1-luc and ISRE-Luc in the EPC cells after overexpression of Myc-*irf3* or Myc-*irf7* and Flag-*plaat1*. As shown in **Figures 4A, B**, overexpression of *irf3* or *irf7* significantly enhanced the promoter activity of IFNφ1 and ISRE, whereas the inducible effects could be abrogated by the overexpression of *plaat1*. Notably, the inhibitory effects were dependent on the doses of *plaat1* plasmid transfected (**Figures 4C–F**). These data suggest that *plaat1* might target *irf3* and *irf7* to negatively regulate the IFN response.

### PLAAT1 Interacts with IRF3 and IRF7

**Figure 5:**
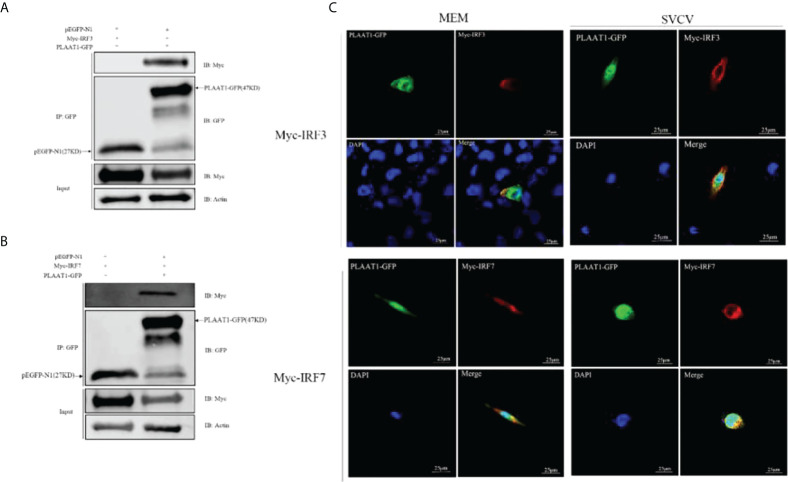
PLAAT1 interacts with IRF3 and IRF7. **(A, B)** EPC cells were transfected with the indicated plasmids (5 *μ*g each). After 24 h, cell lysates were immunoprecipitated (IP) with α-GFP affinity resin. The immunoprecipitates and cell lysates were analyzed by immunoblotting (IB). **(C)** PLAAT1 co-localized with IRF3 and IRF7 in the cytoplasm. with or without SVCV infection. EPC cells were plated onto coverslips in 6-well plates and transfected with PLAAT1-GFP (2 *μ*g) and Myc-IRF3 (2 *μ*g) or Myc-IRF7 (2 *μ*g) plasmids. After 24 h, the cells were left untreated (MEM), infected with SVCV. After an additional 24 h, cells were stained with DAPI (blue) and photographed under a confocal microscope. Green and red colors indicate overexpressed PLAAT1 and IRF3 or IRF7, respectively. Scale bar=25 μm. All experiments were repeated at least three times with similar results.

To explore whether PLAAT1 interacts with IRF3 and IRF7, EPC cells were co-transfected with PLAAT1-GFP and plasmids expressing Myc-IRF3 and Myc-IRF7, and co-IP assays were performed using α-GFP beads. The results showed that the α-GFP Ab-immunoprecipitated protein complexes contained IRF3 and IRF7 (**Figures 5A, B**), revealing that PLAAT1 interacted with IRF3 and IRF7 through protein interaction. Confocal microscopic analysis revealed that the PLAAT1-GFP protein was distributed in the cytoplasm and nucleus with or without SVCV infection (**Figure 5C**). To further determine whether PLAAT1 with IRF3 and IRF7 shared similar subcellular locations. We co-transfected Myc-IRF3 or Myc-IRF7 with PLAAT1-GFP. As shown in **Figure 5D**, PLAAT1 colocalized with IRF3 and IRF7 without or with SVCV infection. Interestingly, SVCV infection resulted in aggregation of PLAAT1 in the cytoplasm. Collectively, the results indicate that PLAAT1 interacts with IRF3 and IRF7.

### PLAAT1 interacts with the IAD of IRF3 and IRF7

**Figure 6:**
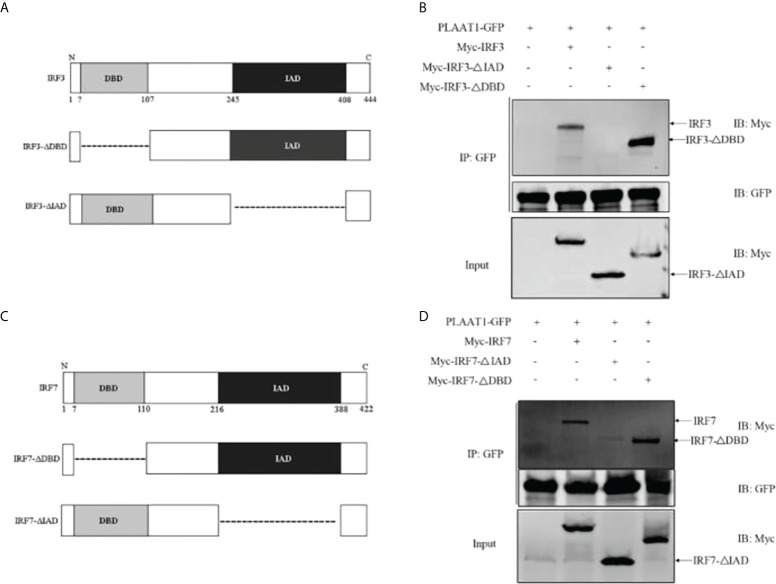
PLAAT1 interacts with the IAD of IRF3 and IRF7. **(A)** Schematic description of IRF3, IRF3-ΔDBD and IRF3-ΔIAD. **(B)** PLAAT1 was immunoprecipitated with IRF3, IRF3-ΔDBD. **(C)** Schematic description of IRF7, IRF7-ΔDBD and IRF3-ΔIAD. **(D)** PLAAT1 was immunoprecipitated with IRF7 and IRF7-ΔDBD. EPC cells were transfected with the indicated plasmids (5 *μ*g each). At 24 h, cell lysates were analyzed by immunoblotting (IB) with the respective Abs.

To characterize the functional domains of IRF3 and IRF7 which are targeted by PLAAT1, we constructed two domain mutants of IRF3 (NCBI accession number: NP_001137376), IRF3-ΔDBD (lacking the DNA-binding domain (DBD), 7-107 aa) and IRF3-ΔIAD (lacking the IRF-association domain (IAD), 245-408 aa) (**Figure 6A**). Co-IP assays were performed to determine the interaction of the two domains with PLAAT1. As shown in **Figure 6B**, the wild type IRF3 and IRF3-ΔDBD were shown to bind to PLAAT1 whereas the IRF3-ΔIAD did not bind, indicating that the IAD but not DBD is required for the interaction between IRF3 and PLAAT1. Similarly, two domain mutants of IRF7 (NCBI accession number: NP_956971) were constructed, IRF7-ΔDBD (lacking the DBD, 7-110 aa) and IRF7-ΔIAD (lacking the IAD, 216-388 aa) (**Figure 6C**). Consistent with the observations in the IRF3/PLAAT1 co-IP assay, the wild type IRF7 and IRF7-ΔDBD were co-immunoprecipitated with PLAAT1 but not the IRF7-ΔIAD (**Figure 6D**). Collectively, these data indicate that PLAAT1 interacts with the IAD but not DBD of IRF3 and IRF7.

### PLAAT1 mediates autophagic degradation of IRF3 and IRF7

**Figure 7:**
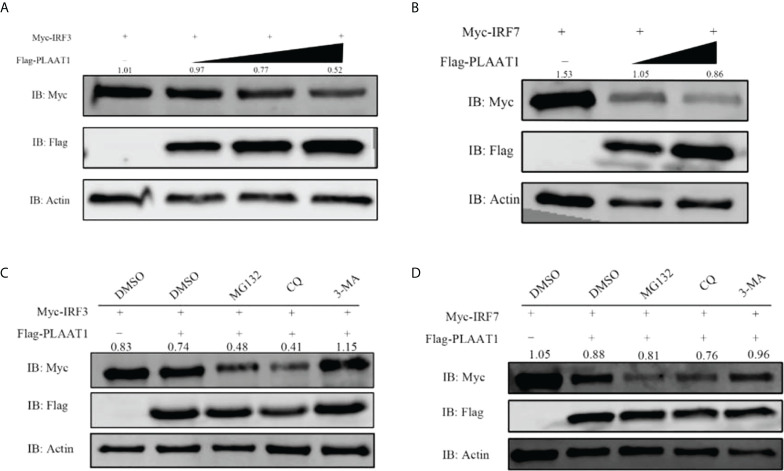
PLAAT1 promotes degradation of IRF3 and IRF7. **(A, B)** Overexpression of PLAAT1 initiated degradation of IRF3 and IRF7 in a dose-dependent manner. EPC cells were transfected with Myc-IRF3 or Myc-IRF7 (1 *μ*g) plus pcDNA3.1 (1 *μ*g) or different amount of Flag-PLAAT1 plasmid (0.5, 1 and 2 *μ*g). Cells were analyzed after 24 h by IB. **(C, D)** 3-MA inhibits PLAAT1 induced degradation of IRF3 and IRF7. EPC cells were transfected with Myc-IRF3 (1 *μ*g) or Myc-IRF7 (1 *μ*g) plus Flag-PLAAT1 (1 *μ*g) or pcDNA3.1 (1 *μ*g). At 18 h, cells were treated with dimethyl sulfoxide (DMSO)(control), MG132 (25 *μ*M), 3-MA (10 mM) or CQ (50 *μ*M) for 6 h, and analyzed by IB. The numbers above the images indicate the ratio of the density of protein bands of IRF3 or IRF7 against that of β-actin.

Given that PLAAT1 interacts with IRF3 and IRF7 and that it suppresses IFN response, we reason that formation of PLAAT1/IRF3 or PLAAT1/IRF7 complex may initiate degradation of IRF3 and IRF7 proteins. For this, we overexpressed the *plaat1* gene together with the *irf3* or *irf7* gene in the EPC cells and observed that PLAAT1 promoted degradation of IRF3 and IRF7 in a dose dependent manner (**Figures 7A, B**). It has been well established that protein is degraded mainly *via* three different pathways involving lysosome, ubiquitin–proteasome and autophagosome. To determine the degradation pathways, we analyzed the effects of pathway inhibitors on the PLAAT1 mediated IRF3/IRF7 degradation. These included chloroquine (CQ, inhibitor of late-phase lysosome dependent autophagy, MG132 (inhibitor of the ubiquitin–proteasome system) and 3-methyladenine (3-MA) (inhibitor of autophagosome). It was shown that the PLAAT1-mediated degradation of IRF3 and IRF7 could be restored by 3-MA but not CQ or MG132 (**Figures 7C, D**), suggesting that overexpression of PLAAT1 could activate degradation of IRF3 and IRF7 *via* autophagosome mediated pathway.

## Discussion

The PLAAT family consists of multiple members that are evolutionary conserved and possess multiple functions. In humans, 5 members are present whereas in mice, 3 are found. All the members possess phospholipase A/acyltransferase activities and play key roles in modifying phospholipids (21). However, the functions of these enzymes are not fully understood. Recently, it has been shown that PLAAT1 and PLAAT3 are involved in degradation of cellular organelles such as mitochondria, endoplasmic reticulum and lysosomes in the lens (33). Moreover, some members of the PLAAT family have been implicated in regulating immune responses. For example, PLAAT3 (also known as PLA2G16) causes membrane rupture of endo-lysosomes to release the viral genomes upon infection with picornavirus and acts as a switch in virus entry and clearance (28). *Plaat3* can be induced by IFN-γ and promotes the premature egress of parasites in humans, hence restricting *Toxoplasma gondii* infection (36). In a previous study, we found that PLAAT1 interacts with p53 and is involved in autophagy (34). Our current work has shown that PLAAT1 inhibits virus-induced production of type I IFN by degradation of IRF3 and IRF7. These results demonstrate that PLAAT1 regulates host immune response against infection.

Interferon response is essential for the host to defend virus invasion. However, the actions of type I IFN must be tightly controlled to avoid excessive IFN response which is detrimental to the host. IRF3 and IRF7 are the master transcription factors driving IFN production in response to viral infection (35). Upon activation by the viral PAMPs, PRRs trigger a cascade of signaling events, leading to phosphorylation of IRF3 and IRF7 and subsequent translocation into nucleus to induce IFN expression (37). This process can be regulated at the multiple levels by host cellular factors and viral proteins. For example, zebrafish sirt7 negatively regulates antiviral response through attenuating phosphorylation of IRF3 and IRF7 (38). The SVCV P protein functions as a TBK1 substrate to decrease IRF3 phosphorylation, reducing IFN transcription and facilitating viral replication (39). On the other hand, the availability of IRF3 and IRF7 for phosphorylation is critical and can be affected by cellular factors mediating protein synthesis and degradation. It has been shown that proteasomal degradation of IRF3 and IRF7 is enhanced by F-box protein fbxo3 in zebrafish, thus inhibiting the IFN production during viral infection (40). In supporting this notion, we show here that PLAAT1 interacts with IRF3 and IRF7, triggering their degradation to mitigate IFN expression. This negative regulation of IFN response could be important to fine-tune the IFN response to restore the hemostasis state after clearance of viruses. On the other hand, viruses could exploit this mechanism to counteract the IFN activated antiviral response.

Protein degradation is regulated *via* three different pathways, engaging the lysosome, ubiquitin-proteasome and autophagosome. Previous studies have suggested that zebrafish IRF3 or IRF7 are regulated by the ubiquitin proteasome pathway, for instance, Uba1 promotes the K48-linked ubiquitination of IRF3, leading to their proteasomal degradation (41). In addition, ovarian tumor domain-containing 6B protein diminishes TRAF6-mediated K63-linked polyubiquitination of IRF3 and IRF7 to suppress IFN production (42). Autophagy also contributes to the regulation of IRF3-mediated antiviral signaling. IFN-induced transmembrane protein 3 (IFITM3) mediates degradation of IRF3 *via* autophagosome-dependent pathway, inhibiting virus-triggered IFN induction (43). Autophagy allows cells to degrade proteins, protein complexes, and organelles through a lysosome-dependent mechanism (44). We demonstrate here that PLAAT1 interacts with IRF3 and IRF7, resulting in their degradation likely through an autophagosome-dependent pathway since 3-MA, an inhibitor of autophagosome, could abolish the inhibitory effect of PLAAT1 on degradation of IRF3 and IRF7 (**Figure 7**). We further show that the interaction between PLAAT1 and IRF3/IRF7 involves the IAD but not the DBD domains of both IRF3 and IRF7. Previous studies have shown that the IADs of IRFs are responsible for the interactions with other proteins including other members of the IRF family (13, 17).

In summary, we show that zebrafish PLAAT1 interacts with IRF3 and IRF7 and triggers degradation of IRF3 and IRF7, therefore blocking IFN production. Our findings reveal a novel role of PLAAT1 in regulating host cellular antiviral response.

## Data availability statement

The raw data supporting the conclusions of this article will be made available by the authors, without undue reservation.

## Author contributions

XZ and WH: investigation, methodology, data curation, and writing original draft. YS, JG, HX, NJ, JF and HD: investigation and methodology. JZ: conceptualization, funding acquisition, project administration, supervision and editing. All authors contributed to the article and approved the submitted version.

## Funding

This work is funded by the National Key R&D Program of China (Grant number: 2018YFD0900302), National Natural Science Foundation of China (Grant numbers: 32030112 and U21A20268) and Key Laboratory of Marine Biotechnology of Fujian Province (Grant Number: 2021MB01).

## Acknowledgments

We thank Dr. Mingxian Chang, Institute of Hydrobiology, Chinese Academy of Sciences, for providing SVCV and EPC cell line.

## Conflict of interest

The authors declare that the research was conducted in the absence of any commercial or financial relationships that could be construed as a potential conflict of interest.

## Publisher’s note

All claims expressed in this article are solely those of the authors and do not necessarily represent those of their affiliated organizations, or those of the publisher, the editors and the reviewers. Any product that may be evaluated in this article, or claim that may be made by its manufacturer, is not guaranteed or endorsed by the publisher.
